# An interdisciplinary intervention for children with complex health complaints; a feasibility study of selection criteria

**DOI:** 10.3389/fped.2023.1167528

**Published:** 2023-09-14

**Authors:** Irene Elgen, Ragnhild B Lygre, Ånen Årli, Torhild Heggestad

**Affiliations:** ^1^ Department of Child and Adolescent Psychiatry, Division of Psychiatry, Haukeland University Hospital, Bergen, Norway; ^2^ Department of Clinical Medicine, University of Bergen, Bergen, Norway; ^3^ Department of Child and Adolescent Medicine, Haukeland University Hospital, Bergen, Norway; ^4^ Department of Research and Development, Haukeland University Hospital, Bergen, Norway

**Keywords:** child health services, patient care, mental health, complex health complaint, psychosomatics

## Abstract

**Background:**

There is a need for re-designing the health service for children suffering from complex and compound health complaints. Based on a previous register study, we have developed criteria to select patients with complex health complaints eligible for an Intervention with an interdisciplinary professional team. The team consists of a pediatrician, a psychologist and a physiotherapist.

**Method:**

To identify children with complex health complaints who would benefit from this intervention, we have selected a group of patients by using a set of criteria consisting of the following criteria: multi-referred young school age children referred to both mental health service and pediatric service. This study focuses on the feasibility of these criteria by measuring participation and compliance and by gathering feedback from the team members in the interdisciplinary team.

**Results:**

Among 677 children aged 6−12 years referred to a regional hospital, we found 32 (5%) children eligible for the interdisciplinary Intervention according to the applied criteria. Only 6% of the invited parents declined to participate in the intervention. According to the interdisciplinary team, the intervention was found suitable for 88% of the children.

**Conclusion:**

The suggested criteria seemed feasible, in terms of identifying eligible patients for this interdisciplinary Intervention for children with complex health complaints.

**Clinical Trial Registration:**

Retrospectively registered on www.clinicaltrials.gov, ID NCT04652154, on the 3rd of December 2020.

## Key messages regarding feasibility

1. What uncertainties existed regarding the feasibility?
  a) Are the suggested inclusion criteria feasible for identifying the target group?
  b) Will the child and family accept meeting not only a medical doctor, but a whole professional team?
  c) Do the professional team regard this intervention as useful for the selected families?
2. What are the key feasibility findings?
  a) The criteria for offering an interdisciplinary Intervention for children with complex health complaints were found suitable and feasible, with a positive predictive value of 0.88.
  b) 27 out of 32 (84%) eligible patients consented to participate in the study.
  c) The professional team evaluated the intervention as suitable for 88% of the patients completing the Intervention, with a positive predictive value of 0.88.
3. What are the implications of the feasibility findings for the design of the main study?
  a) The selections criteria are feasible for a case-control study.
  b) The main study should expect about 80% of eligible patients to consent to participating in the study, and thus 20% attrition from assessed eligibility to allocation.
  c) The Intervention may have little effect for about 10%–20% of the families completing the Intervention.

## 1. Background

Children with repeated referrals to different medical specialities due to complex health complaints pose not only a diagnostic challenge, but also raise questions on how to organize the specialist health service (1–4). Such complex health complaints encompass compound and diffuse symptoms difficult to disentangle, assess and treat effectively. Most common is stomach- and headache with low energy and attention problems, not attributable to a specific illness. To improve and simplify the time-consuming and expensive diagnostic process, there has been a call for a re-designing of health services (5–9).

To increase our understanding of children with complex health complaints, a register study investigated more than 18,000 referrals to a regional hospital of children six to twelve years of age (2). A high rate of non-specific diagnoses was found, as well as complex care patterns, particularly for patients with three or more referrals, including to both mental health service and paediatric clinics. The complexity of these suggest that there is a need to simplify care patterns and increase collaboration between different hospital departments (10), to reduce fragmented care and promote a more holistic/interdisciplinary health service. This could prevent repetitive diagnostic testing and different symptom explanations (7, 8, 11–17).

However, the register study also confirmed that it is difficult to find a single discriminating factor that would identify the specific group of children who are most in need of a more integrative and interdisciplinary healthcare approach (2). To further explore this question, the register study was supplemented with an audit study of 250 children (5). For 15% of multi-referred school-age children, the diagnostic process ended without a specific conclusion and treatment plan. The pediatric sub-specialties most often included in such multiple referrals were found to be gastroenterology and neurology. The retrospective studies recommended innovations re-designing healthcare for children with complex care patterns, to reduce the fragmentation of care that these children are at risk of being afflicted by (5).

An interdisciplinary Intervention has been developed and piloted, and different challenges in re-designing the health service have been addressed (18). In short, school age children with previous multi-referrals, and with the latest somatic referral to either gastro-enterology or neurology, were included in the pilot study, and offered the interdisciplinary Intervention. The children were assessed by a professional team consisting of a psychologist, a paediatrician and a physiotherapist in a two to two and a half hours long interdisciplinary consultation. The intent of this interdisciplinary Intervention is to attempt to clarify the child's condition. However, the criteria for inclusion of young school age children in such an interdisciplinary Intervention have not previously been systematically evaluated.

The aims of the present study were:

1) To test the suggested inclusion criteria
2) To evaluate how acceptable it was for a patient and its family to participate in a interdisciplinary Intervention
3) To test the suitability and feasibility of the intervention for the invited children and parents, as evaluated by the professional team-members.

## 2. Methods

### 2.1. Intervention

Haukeland University Hospital is a regional hospital providing healthcare to children across a wide range of clinical specialties, including mental health. Its catchment area covers a population of about half a million inhabitants and serves a regional population of one million.

The intervention consists of an interdisciplinary Intervention including a pediatrician, psychologist and physiotherapist, and has been described in detail previously (18). The Intervention starts with patient and parents sharing their concerns and health complaints to the joint team. Then the professionals discuss within the team and customize a plan for the rest of the intervention, assess what further information is needed and what should be the focus of the Intervention. Then the assessment takes place. This part is tailored individually for each patient and could include further interviews and/or clinical examination. Given the heterogeneous nature of the group of patients, no standardized approach is given. Before rounding up, the professionals summarizes the assessment within the team. Finally, the joint team, including the child and its parents, makes a shared decision on how to understand the condition, if further clinical examinations are needed, and establish a treatment plan (5).

### 2.2. Inclusion criteria and process

Parents of selected children fulfilling the inclusion criteria were invited to the study by a phone call from our research nurse. They were informed that the hospital had received a referral from the family doctor to a pediatrician; however, the medical record indicated need of a more interdisciplinary approach. The parents could decide whether they wanted to participate in the study or follow the standard procedure meeting only a pediatrician. No promise was given as to shorter waiting times than the standard care. Written informed consent was obtained on the day of the Intervention.

**Figure 1:**
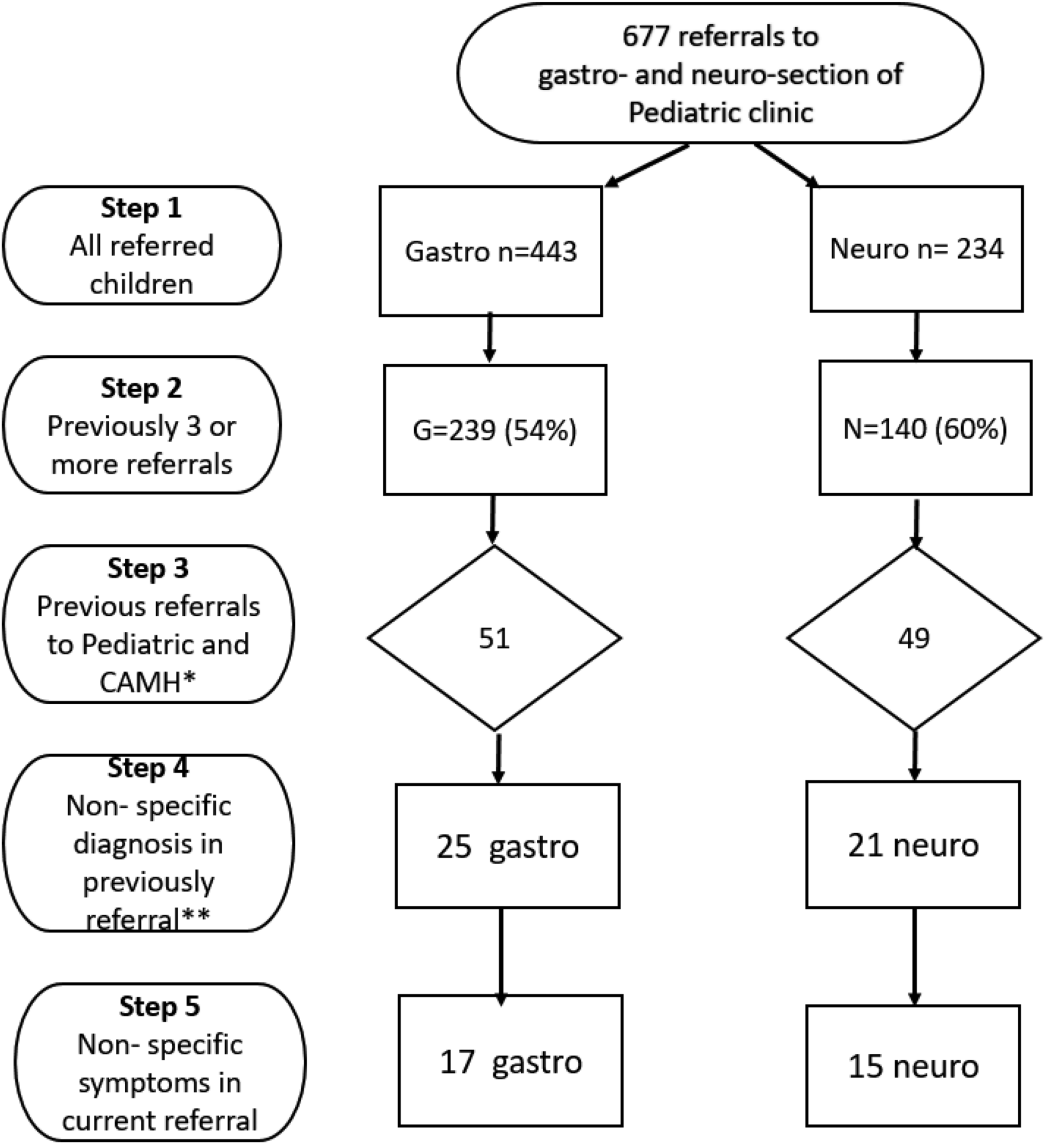
Steps for selecting children with complex health complaints to an interdisciplinary intervention.

The steps for selecting children with complex health complaints eligible for an interdisciplinary Intervention are shown in Figure 1.

• Total number of children referred to outpatient specialist health care services for symptoms related to the nervous or gastrointestinal systems (Step 1).
• Children with three or more previous referrals within the last three years, with at least one referral to child and adolescent mental health service (CAMHS) and one to the pediatric department (Step 2 + 3).
• The previous referrals were examined to see whether the last diagnose was an unspecified diagnose or not (Step 4).
• The current referral was further medically assessed to find whether the actual health complaint(s) could be considered an unclear or compound condition, and thus in need of an alternative intervention (Step 5).

Examples of issues that was considered important for the assessment in step 5: (1) Previous assessment for the same symptom has been inadequate; (2) The health complaints presented were diffuse and not related to a chronic illness; (3) Underlying issues like school refusal or conflicts within the family when such problems were addressed in the referral letter. This assessment was performed solely based on information from the referral letter and the patient's medical record by a senior clinician (IBE).

Step 1–4 could well be assessed by applying an algorithm be digitally processed. To perform step 5, an assessment using a qualified medical professional was absolutely needed. We wanted to estimate the positive predictive value of the selection process by asking the health professionals about their experiences.

### 2.3. Acceptability/participation and evaluation

#### 2.3.1. Acceptability/participation

For all children meeting the inclusion criteria, data was obtained regarding consent to participate, the occurrence and reasons of drop outs during the intervention, and finally, the number of children and parents completing the Intervention. These data were defined as a measure of acceptability for participating in the re-designed health service.

#### 2.3.2. Professional team evaluation

After the interdisciplinary Intervention the professional teams were invited to evaluate the intervention in terms of suitability and usefulness for the child and family. In addition, the feasibility was further explored by assessing if the condition was resolved, and if the professional team and parents ended up with a joint understanding of the condition. The team completed a questionnaire to answer the questions specified in Table 1. The team were also asked to suggest the possibility/feasibility of introducing other inclusion criteria.

**Table 1:** Professional team evaluation of the utility of the interdisciplinary intervention for the selected children. *N* = 24.

Question	Measures
*Suitability* The intervention today was useful for the child *1 = not at all 5 = Very useful*	4.1 (SD:0.9)
The intervention today was useful for the parents *1 = not at all 5 = Very useful*	4.3 (SD:0.6)
The intervention was suitable for the child	21 (88%)
*Feasibility* The condition was resolved	9 (38%) resolved 10 (42%) resolved after one follow up 4 (17%) diagnosed with chronic illness 1 (4%) unresolved
Professional team and parents agreed upon the condition	23 (96%) agreement

### 2.4. Statistical analyses

For descriptive analyses, we used mean scores and standard deviation (SD). Positive Predictive Value of the inclusion criteria was calculated from the formula True positives/(True positives + False positives). The SPSS statistical package version 24.0 was used for analyses (19).

## 3. Results

### 3.1. Inclusion process

In 2020 the included paediatric sections at the hospital had 677 referrals of children age six to 12 years to the outpatient clinic. The criterias are described (Figure 1). For multi-referred children 100/677 (15%) met the first three selection criteria, and 46/677 (7%) had a non-specific diagnose at the last visit to the hospital. When further evaluating the child's presenting medical condition by the trained clinician, we found 32 out of 677 (5%) with non-specific referral symptoms and meeting all the selection criteria. Comparing the criteria given in steps 1–4, where the selection can be performed by a simple algorithm, with the result after the 5th professional assessment, gave a Positive predictive value (PPV) of 0.7 (32/46).

### 3.2. Acceptability/participation

For 32 eligible patients 27 (84%) gave written consent to participate in the study. However, two were hospitalized before the intervention, due to circumstances not related to their referral, and therefore excluded. Regarding the patients not consenting, two patients were already in a treatment process when being offered the study intervention, and one had moved. Two patients (6%) declined the invitation, leaving us with 25 (78%) patients. All 25 families completed the Intervention and gave consent to a follow up after one year.

### 3.3. Professional team evaluation

For the 25 patients completing the intervention, the professional team evaluated 24 of the Interventions (Table 1). Twenty one patients (88%) were evaluated as suitable for the intervention. Using the result of 3 false positives of 24 to calculate the PPV, gave the result 21/24 = 0.88.

As to the effect, the condition was resolved during the session for nine (38%) patients, who were not in need of further specialist health care. For 10 (42%) patients, their condition was clarified through the Intervention, however, they were considered to need at least one follow-up Intervention to ensure the resolvement. Four patients were diagnosed with a chronic illness, and two of these were found to benefit from an interdisciplinary Intervention in order to assess the complexity of the chronic condition.

In sum the evaluation from the professional team suggested that only three of the patients did not benefit from the new intervention. One patient did not benefit from the intervention probably due to high level of conflict between the parents. For two patients the professional team was uncertain regarding the feasibility, as these two did not seem to agree on treatment plans, even though they agreed on a joint understanding of the condition. Both patients had chronic illnesses.

The professional team found that school refusal was often described in the referral letter and they recommended this issue as an additional inclusion criteria.

## 4. Discussion

In this feasibility study, the criteria for selecting patients to a professional Intervention were evaluated. The chosen criteria were found to have a positive predictive value of 0.88 for children with complex health complaints in need of an interdisciplinary approach. Furthermore, being invited to assessment from an interdisciplinary team was accepted by almost all children and families. The professionals assessing the Intervention found that seven in eight children seemed to benefit from it.

### 4.1. Inclusion criteria

As this patient group is a heterogeneous one with a complex set of symptoms, an evaluation of inclusion criteria is important. For this feasibility study it was important to ensure a low number of “false positives”. This was achieved particularly by adding a last assessing step, where the actual condition was specifically addressed, in addition to the history, which could easily be reached by a simple algorithm.

### 4.2. Acceptability

When evaluating the feasibility of this new intervention, the first step was to make sure that the child and family would accept meeting not only a medical doctor, but a whole professional team. Most of the patients and families gave written consent to participate in the study and completed the Intervention, giving support to good compliance. Further, even though the child primarily was referred to a standard 20–30 min Intervention with a pediatrician at the hospital, the families accepted an alternative option that also included a physiotherapist and a psychologist, and that implied using half a day at the hospital. It is important to obtain the experiences of child and parents before continuing the process towards a case-control study of the intervention.

### 4.3. Team evaluation

The professional team regarded the intervention as useful in most cases. As described in a previous paper the professional teams has received coaching on working as an interdisciplinary team (5). We suggest that the team interaction is an important factor towards successful collaboration with the child and family helping them to a better understanding of the child's condition and complaints. Among the participants a great number of children had functional impairments, i.e., school refusal. The professionals recommended that school refusal should be evaluated as a supplementary inclusion criteria in future studies.

### 4.4. Strengths and limitations

The strength of the present study is the thoroughly and systematic register exploration of all children referred to a regional hospital over a period of time. Testing the inclusion criteria for this diagnostically heterogeneous group of children, is important. A limitation of this study was the low number of participants. However the results are in line with the initial retrospective register study, in terms of the number of identified cases in this population (2), thus the sample is expected to be representative of the population. From the 677 children referred in the period, only five percent was found to meet the chosen criteria. One could ask if these criteria were too strict, and if the suggestion of including school refusal as an alternative criteria (either/or) to being referred to both pediatrics and CAMHS, would increase the number of children benefitting from meeting an interdisciplinary team. However, for this pilot it was important to ensure a low number of “false positives” and let the upcoming evaluation of outcome measures guide further choices. Furthermore, it is important to evaluate child and parents experiences with compound health complaints.

## 5. Conclusion

In this study the criteria for offering an interdisciplinary Intervention to young school age children with complex health complaints, were found suitable and feasible to identify those children in need of an interdisciplinary assessment. The results support a further evaluation of the interdisciplinary Intervention through a case-controlled study.

## Data Availability

The original contributions presented in the study are included in the article, further inquiries can be directed to the corresponding author.

## Trial Registration

Data was obtained from Haukeland university hospital the patient registry system No. 2017/12470. Start of registration was January 1st 2020 and patients included was from 2020 to 21.

Transitioning Young Patients’ Health Care Trajectories*, NCT04652154. Registered December 3rd, 2020 - Retrospectively registered,* https://clinicaltrials.gov/ct2/show/NCT04652154?term=NCT04652154&draw=2&rank=1.
